# Draft Genome Sequences of *Pseudomonas fluorescens* BS2 and *Pusillimonas noertemannii* BS8, Soil Bacteria That Cooperate To Degrade the Poly-γ-d-Glutamic Acid Anthrax Capsule

**DOI:** 10.1128/genomeA.00057-12

**Published:** 2013-01-24

**Authors:** Richard A. Stabler, David Negus, Arnab Pain, Peter W. Taylor

**Affiliations:** a London School of Hygiene and Tropical Medicine, London, United Kingdom; b University College London, School of Pharmacy, London, United Kingdom; c King Abdullah University of Science and Technology, Thuwal-Jeddah, Saudi Arabia

## Abstract

A mixed culture of *Pseudomonas fluorescens* BS2 and *Pusillimonas noertemannii* BS8 degraded poly-γ-d-glutamic acid; when the 2 strains were cultured separately, no hydrolytic activity was apparent. Here we report the draft genome sequences of both soil isolates.

## GENOME ANNOUNCEMENT

Experimental infections caused by bacteria that elaborate a polysaccharide or polypeptide capsule can be resolved by administration of capsule hydrolases that strip away the protective capsular layer from the bacterial surface (1, 2). Anthrax is an excellent candidate for this approach: the inhalational form of the infection requires prompt therapy, and the causative agent, *Bacillus anthracis*, invariably expresses a unique poly-γ-d-glutamic acid (PDGA) capsule that is necessary for optimal pathogenesis (3). If successful, the capsule-stripping approach could confound attempts to render anthrax untreatable by the engineering of multidrug-resistant strains for unlawful dissemination. The poly-γ-glutamate-specific capsule depolymerase (CapD) produced by *B. anthracis* removes the capsule from the surface of the anthrax bacillus (4) and can protect against anthrax infection (5) but is markedly unstable (6) and therefore unlikely to be useful for therapeutic development.

We have recently obtained a highly stable PDGA-specific capsule depolymerase from bacterial cultures (unpublished data); soil enrichment techniques were employed to isolate bacteria with the capacity to degrade PDGA and to use the hydrolytic products as the sole source of carbon and energy. The most pronounced hydrolytic activity was associated with a strongly mutualistic consortium culture comprising two morphologically distinct colony types; when the two strains were cultured separately, no depolymerase activity was apparent in either culture. A 16S sequence identified these organisms as *Pseudomonas fluorescens* (s_ab score = 1) and *Pusillimonas noertemannii* (s_ab score = 0.996). The two isolates, *P. fluorescens* BS2 and *P. noertemannii* BS8, were sequenced in order to provide a database for the identification of the PDGA depolymerase and to provide a platform for elucidation of the basis of interstrain cooperation. Only one *Pusillimonas* species genome sequence, from an oil-degrading bacterium isolated from the Bohai Sea, is currently available (7).

Whole-genome sequencing was performed on the Illumina HiSeq 2000 platform using a single read (read 1) from a paired-end read library with read lengths of 100 bp. The short sequence reads were first processed with Trimmomatic (8) and quality assessed using FastQC (http://www.bioinformatics.babraham.ac.uk//projects/fastqc/) software. VelvetOptimiser (http://bioinformatics.net.au/software.velvetoptimiser.shtml) was used for optimization of the Velvet *de novo* assembly (9), resulting in 122 contigs with an *N*_50_ of 5,355 bp for *P. fluorescens* BS2, comprising in total 6,123,259 bp, and 121 contigs with an *N*_50_ of 15,214 bp for *P. noertemannii* BS8, comprising in total 3,916,977 bp. Automated gene prediction and annotation were performed using RAST (10), which predicted 5,539 and 3,633 coding sequences for *P. fluorescens* BS2 and *P. noertemannii* BS8, respectively. Interestingly, RAST indicated that the nearest neighbor to *P. fluorescens* BS2 was *P. fluorescens* SBW25 and to *P. noertemannii* BS8 was *Bordetella bronchiseptica* RB50.

### Nucleotide sequence accession numbers.

The *Pseudomonas fluorescens* BS2 and *Pusillimonas noertemannii* BS8 sequences have been deposited in NCBI under accession numbers AMZF00000000 and AMZG00000000. Short reads have been deposited in the SRA under accession number SRA058672.

## References

[1] Mushtaq N Redpath MB Luzio JP Taylor PW 2004 Prevention and cure of systemic *Escherichia coli* K1 infection by modification of the bacterial phenotype. Antimicrob. Agents Chemother. 48(5):1503–1508 15105097 10.1128/AAC.48.5.1503-1508.2004 PMC400570

[2] Taylor PW Bernal P Zelmer A 2009 Modification of the bacterial phenotype as an approach to counter the emergence of multidrug-resistant pathogens, p 43–78 Bonilla AR Muniz KP, Antibiotic resistance: causes and risk factors, mechanisms and alternatives. Nova Science Publishers, Hauppauge, NY

[3] Mock M Fouet A 2001 Anthrax. Annu. Rev. Microbiol. 55:647–671 11544370 10.1146/annurev.micro.55.1.647

[4] Scorpio A Chabot DJ Day WA O’Brien DK Vietri NJ Itoh Y Mohamadzadeh M Friedlander AM 2007 Poly-gamma-glutamate capsule-degrading enzyme treatment enhances phagocytosis and killing of encapsulated *Bacillus anthracis*. Antimicrob. Agents Chemother. 51(1):215–222 17074794 10.1128/AAC.00706-06 PMC1797643

[5] Scorpio A Tobery SA Ribot WJ Friedlander AM 2008 Treatment of experimental anthrax with recombinant capsule depolymerase. Antimicrob. Agents Chemother. 52(3):1014–1020 18160516 10.1128/AAC.00741-07 PMC2258529

[6] Wu SJ Eiben CB Carra JH Huang I Zong D Lui P Wu CT Nivala J Dunbar J Huber T Senft J Schokman R Smith MD Mills JH Friedlander AM Baker D Siegel JB 2011 Improvement of a potential anthrax therapeutic by computational protein design. J. Biol. Chem. 286(37):32586–32592 21768086 10.1074/jbc.M111.251041 PMC3173206

[7] Cao B Ma T Ren Yan Ren Yi Li G Li P Guo X Ding P Feng L 2011 Complete genome sequence of *Pusillimonas* sp. T7-7, a cold-tolerant diesel oil-degrading bacterium isolated from the Bohai Sea in China. J. Bacteriol. 193:4021–4022 21622753 10.1128/JB.05242-11 PMC3147516

[8] Lohse M Bolger AM Nagel A Fernie AR Lunn JE Stitt M Usadel B 2012 Robina: a user-friendly, integrated software solution for RNA-Seq-based transcriptomics. Nucleic Acids Res. 40 (Web Server issue):W622–W627. doi: 10.1093/nar/gks540 PubMed 10.1093/nar/gks540 PMC3394330 22684630

[9] Zerbino DR Birney E 2008 Velvet: algorithms for de novo short read assembly using de Bruijn graphs. Genome Res. 18(5):821–829 18349386 10.1101/gr.074492.107 PMC2336801

[10] Aziz RK Bartels D Best AA DeJongh M Disz T Edwards RA Formsma K Gerdes S Glass EM Kubal M Meyer F Olsen GJ Olson R Osterman AL Overbeek RA McNeil LK Paarmann D Paczian T Parrello B Pusch GD Reich C Stevens R Vassieva O Vonstein V Wilke A Zagnitko O 2008 The RAST server: rapid annotations using subsystems technology. BMC Genomics 9:75 18261238 10.1186/1471-2164-9-75 PMC2265698

